# How Much of Me Do I See in Other Minds? Modulating Egocentricity in Emotion Judgments by tDCS

**DOI:** 10.3390/brainsci11040512

**Published:** 2021-04-16

**Authors:** Anne Weigand, Irene Trilla, Lioba Enk, Garret O’Connell, Kristin Prehn, Timothy R. Brick, Isabel Dziobek

**Affiliations:** 1 Berlin School of Mind and Brain, Humboldt-Universität zu Berlin, Unter den Linden 6, 10099 Berlin, Germany; irene.trilla@hu-berlin.de (I.T.); enk.lioba@gmail.com (L.E.); garretoconnell1@gmail.com (G.O.); isabel.dziobek@hu-berlin.de (I.D.); 2 Department of Psychology, Humboldt-Universität zu Berlin, Unter den Linden 6, 10099 Berlin, Germany; 3 MSH Medical School Hamburg, University of Applied Sciences and Medical University, Am Kaiserkai 1, 20457 Hamburg, Germany; kristin.prehn@medicalschool-hamburg.de; 4 Department of Human Development and Family Studies and Institute for Computational and Data Sciences, The Pennsylvania State University, State College, 231 Health and Human Development Building, University Park, PA 16802, USA; tbrick@psu.edu

**Keywords:** egocentric bias, self–other differentiation, supramarginal gyrus, tDCS, transcranial direct current stimulation

## Abstract

When inferring the mental states of others, individuals’ judgments are influenced by their own state of mind, an effect often referred to as egocentricity. Self–other differentiation is key for an accurate interpretation of other’s mental states, especially when these differ from one’s own states. It has been suggested that the right supramarginal gyrus (rSMG) is causally involved in overcoming egocentricity in the affective domain. In a double-blind randomized study, 47 healthy adults received anodal (1 mA, 20 min) or sham transcranial direct current stimulation (tDCS) to the rSMG prior to performing a newly developed paradigm, the self–other facial emotion judgment (SOFE) task. In this task, participants made judgments of facial emotional expressions while having been previously confronted with congruent or incongruent emotion-inducing situations. To differentiate between emotional and cognitive egocentricity, participants additionally completed an established visual perspective-taking task. Our results confirmed the occurrence of emotional egocentric biases during the SOFE task. No conclusive evidence of a general role of the rSMG in emotional egocentricity was found. However, active as compared to sham tDCS induced descriptively lower egocentric biases when judging incongruent fearful faces, and stronger biases when judging incongruent happy faces, suggesting emotion-specific tDCS effects on egocentric biases. Further, we found significant tDCS effects on cognitive egocentricity. Results of the present study expanded our understanding of emotional egocentricity and point towards emotion-specific patterns of the underlying functionality.

## 1. Introduction

As social beings, humans often rely on the ability to correctly interpret others’ mental states in order to interact successfully. To understand the thoughts and feelings of others, it is necessary to co-activate and alternate between, at times conflicting, representations of the self and others, i.e., to engage in self–other distinction [1]. Difficulties in self–other distinction can result in egocentric biases, which have been investigated by a broad range of experimental paradigms in the cognitive domain [2,3]. Cognitive egocentricity occurs when the own knowledge about a given situation affects inferences about how someone else judges the situation. Accordingly, it can also influence decisions during visual perspective-taking [4], which involves the understanding that one’s own viewpoint (i.e., self) may differ from the viewpoint of another observer (i.e., other).

Self–other distinction is of equal importance to empathy [5,6,7]. To adequately respond to the emotions of others while being in a different emotional state, a person needs to disengage from their own states to represent the other without bias (e.g., when you are thrilled to cross a rope bridge on a hike while the person next to you has an intense fear of heights and is very afraid). A failure in this process can lead to emotional egocentricity, i.e., when the judgment of someone else’s emotional state is biased towards one’s own emotional state [3]. To date, there was only a small number of experimental paradigms tailored to specifically investigate egocentricity in the emotional domain. The first paradigm assessing egocentric biases when inferring others’ emotions was based on the induction of pleasant or unpleasant feelings via visuo-tactile stimulation [8]. In some trials, subjects had to disengage from their own experience of an affective touch (i.e., self) in order to correctly judge the emotional state of another person (i.e., other) undergoing a simultaneous affective touch of incongruent valence. Similar paradigms evoked congruent or incongruent emotions between a participant and another person via visuo-gustatory [9] or audio-visual [10] stimulation or via a monetary reward and punishment manipulation in a competitive game [11].

On a neural level, the right temporoparietal junction (rTPJ) showed to be critically involved in overcoming cognitive egocentricity [12]. Notably, to overcome emotional egocentricity, a specific role of a region located slightly anterior to the rTPJ, namely the right supramarginal gyrus (rSMG), was identified [8]. In line, resting-state networks analyses showed increased functional connectivity of rTPJ to the brain regions involved in cognitive perspective-taking, such as the medial prefrontal cortex and the precuneus, while the rSMG showed increased functional connectivity to brain regions involved in affective empathy such as the anterior insulae and the middle cingulate cortex [13,14].

To expand our understanding of emotional egocentricity and rSMG functioning, we developed a novel emotional egocentricity paradigm, the self–other facial emotion judgment (SOFE) task, and used it in a noninvasive brain stimulation experiment. In contrast to standard egocentricity paradigms, where participants have to imagine the reactions of other persons for an affective stimulation (e.g., after being touched with pleasant versus unpleasant stimuli; [8]), the SOFE task asked participants to evaluate the facial-emotional expressions of other persons in response to emotional situations, which represents a common scenario in everyday life. In our own previous research, we demonstrated the occurrence of egocentric projections when affective inferences relied directly on reading others’ facial expressions [15]. In the SOFE task, we first confronted our participants with situations that could evoke happiness or fear (e.g., jumping out of a plane for a skydive or riding a roller coaster down a steep track) and asked them to rate their own experienced emotion in the respective situation. We then instructed participants to rate the emotional facial expression of another person being in the same situation. As a baseline condition, the same facial stimuli were also rated without a preceding situational stimulus. Biases in emotion judgments were estimated by comparing the emotional ratings of the others’ facial expressions with a situational context vs. without a situational context. Emotional egocentricity was indicated if the presentation of an emotionally-inducing situation biased the emotion judgments towards the participant’s own reactions to that particular situational cue.

Using anodal transcranial direct current stimulation (tDCS), which involved the application of a weak electrical current to enhance brain activity, we aimed to investigate the causal role of rSMG in overcoming emotional egocentricity during SOFE task performance. To further assess the distinct role of rSMG for emotional as compared to cognitive egocentricity, we additionally tested the effects of tDCS applied to the rSMG on the Director task performance, an established measure of egocentricity in visual perspective-taking [16] that had been used in previous tDCS experiments [12,17].

In addition, we explored associations with previously identified potential factors influencing the degree of egocentric tendencies, including perspective-taking tendencies [15] and autistic traits [18,19].

To sum up, we aimed to investigate the causal role of the rSMG in a novel emotional egocentricity paradigm by means of tDCS. Based on previous findings, we hypothesized that anodal tDCS to the rSMG would increase subjects’ ability to overcome emotional egocentricity but would not alter cognitive egocentricity.

## 2. Materials and Methods

### 2.1. Sample

Forty-seven native speakers of German (28 females) with a mean age of 26 ± 3 years (range: 19–30 years) participated in the study for monetary compensation. Study participants had no previous or concomitant neurological or psychiatric illness or any contraindications to tDCS [20]. All subjects were right-handed and naïve to tDCS. The study was approved by the ethics committee of the psychology department at Humboldt-Universität zu Berlin. Written informed consent was obtained prior to study entry in accordance with the Declaration of Helsinki. Pursuant to standard reviewer disclosure requests (see http://osf.io/hadz3, accessed on 11 April 2021), we confirm that this paper reported all measures, conditions, and data exclusions. The sample size was determined using an a priori power analysis conducted in G*Power (v 3.1.9.4; [21]), which yielded a sample size of 46 participants (23 per tDCS condition) using an alpha level of 0.05 and a power of 0.70. for a *η*_p_^2^ = 0.11. The effect size was determined based on emotional egocentricity effects reported in an experiment consisting of 45 participants undergoing repetitive transcranial magnetic stimulation (rTMS) of the rSMG (*η*_p_^2^ = 0.098; [8]).

### 2.2. Experimental Procedure

In a double-blind between-subjects design, participants were randomly assigned to receive either active (*N* = 24) or sham tDCS (*N* = 23) over the rSMG. During stimulation, they performed an adapted version of the Director task [16]. Immediately afterward, participants completed our newly developed emotional egocentricity paradigm, the SOFE, task. Prior to the actual experiment, all subjects underwent a comprehensive familiarization and practice phase of both paradigms. Tasks were programmed and administered in MATLAB (The MathWorks Inc., Natick, MA, USA) using the Psychophysics Toolbox extensions [22,23].

### 2.3. Emotional Egocentricity: SOFE Task

**Figure 1:**
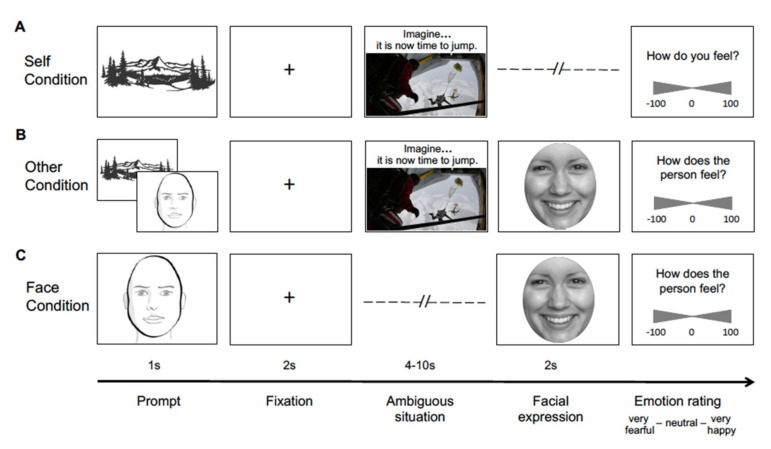
Example stimuli of the SOFE task. Participants were instructed to first imagine themselves in a happy or fearful situation and to either rate their own experienced emotion (Self condition, **A**) or the emotional expression displayed by another person being in the same situation (Other condition, **B**). In the Face condition (**C**), participants were asked to rate the same facial emotional expressions without a preceding situational stimulus. The two-sided continuous rating scale ranged from very fearful (−100) over neutral (0) to very happy (100). Conditions were introduced by brief image prompts.

The SOFE task consists of three conditions: Self, Other, and Face (Figure 1). In the Self and Other conditions, participants were asked to imagine themselves or another person in a particular situation that could elicit either happiness or fear. The situation was presented onscreen for 4–10 s as a photograph depicting an event from a first-person perspective, accompanied by a short, written description of the situation (e.g., jumping out of a plane for a skydive). Immediately after stimulus offset, participants had to either rate their own experienced emotion (Self condition) or the emotion of another person (Other condition) whose facial emotional expression was presented for 2 s. In the Face condition, participants were asked to rate the same facial emotional expressions presented in the Other condition, but this time without a preceding situational stimulus. In all three conditions, participants provided their emotion ratings via mouse click on a two-sided continuous scale ranging from very fearful (−100) over neutral (0) to very happy (100).

In total, the SOFE task consisted of 120 experimental trials (task duration: approximately 40 min), divided into 4 randomized runs of 30 trials each. Conditions were introduced blockwise by brief image prompts (see Figure 1). Each randomized run started with the Self condition (6 trials), followed by the Other condition (12 trials, including 6 situational stimuli, each coupled once with a happy face and once with a fearful face of the same gender) and the Face condition (12 trials, including 6 happy faces and 6 fearful faces), or vice versa. The order of the Other and Face condition blocks was counterbalanced across runs.

The 24 different situational stimuli had been selected in a qualitative pretest out of 50 photographs as representing the most emotionally ambiguous situations (i.e., causing the most polarized responses on the continuum between very fearful and very happy) based on 15 independent ratings (see Table 1 for the selected stimuli). For example, imagining skydiving makes some people generally happy and others generally afraid, while only a few people react emotionally indifferent. Facial stimuli consisted of images of happy and fearful facial expressions of different intensities and identities. Emotion expressions of varying intensity were created by mixing a neutral face with a happy or a fearful face of 4 male and 4 female models taken from a standardized dataset [24]. For each model, morphing was done in steps of 5% along a continuum from 10% to 90% fear or happiness using Winmorph 3.01 (www.debugmode.com/winmorph/, accessed on 19 April 2016). Three happy and 3 fearful morphed faces were randomly selected for each face identity to compose the 48 different emotional faces (24 happy, 24 fearful) presented in the task. Different intensities of emotional expressions were used instead of full-blown expressions to facilitate variability in the emotion ratings. The facial stimuli presented in the Face and Other conditions were identical.

### 2.4. Cognitive Egocentricity: Director Task

The Director task [16] required participants to take into account the viewpoint of a character introduced as the director and to follow his auditory instructions to move objects on a 4 × 4 grid shelf containing eight different objects. Five slots were occluded from the director’s viewpoint, who stood on the opposite side of the shelf. Participants listened to auditory instructions from the director, who asked them to move specific objects in particular directions. Each trial started with the presentation of the shelf for 2 s, followed by the auditory instruction. Instructions were re-recorded into German from the original version [25] and matched for the duration (approx. 2.2 s each). Importantly, some of the objects were placed in occluded slots and therefore only visible to the participants but not to the director (competitor objects). Instructions on experimental trials referred to these competitor objects on the following dimensions: space (e.g., “move the top apple” could refer to a higher hidden apple), size (e.g., “move the small apple” could refer to a larger hidden apple), or semantics (e.g., “move the mouse” could refer to a computer mouse or a hidden toy mouse). Instructions in control trials were identical to experimental trials except for the competitor object being replaced by an object that was irrelevant to the instruction. Reaction times and accuracy of selection and movement of the target objects were recorded. In total, the Director task consisted of 48 trials (task duration: approximately 20 min), divided into 24 experimental and 24 control trials presented in a randomized order.

### 2.5. tDCS Protocol

tDCS was delivered using a battery-driven constant current stimulator (neuroConn DC-STIMULATOR PLUS, Ilmenau, Germany). The anode (5 cm × 7 cm) was positioned over CP4 according to the international 10–20 system for electroencephalography (EEG) electrode placement [26], successfully used in previous studies to place the electrode over the rSMG [27,28]. The cathode (10 cm × 10 cm) was positioned over the contralateral supraorbital region. Participants were assigned to the active or sham tDCS using a randomizing function of the tDCS device. Active tDCS was applied at 1mA for 20 min with a 30 s ramp-up at the beginning and a 30 s ramp-down period at the end of stimulation. Previous studies using similar stimulation parameters showed neuromodulatory effects up to 60 min post-stimulation [29,30]. For the sham condition, a short (one minute) current was delivered, which showed to evoke a similar sensation as receiving active stimulation [31,32] without leading to a neurophysiological change that could influence performance [29]. At the end of the experiment participants were asked to complete a standard questionnaire assessing tDCS adverse effects [33] and to guess whether they had received active or sham tDCS.

### 2.6. Control Variables

To control for interindividual differences in characteristics that might influence the task performances independent of tDCS, participants were asked to complete a series of online questionnaires prior to the experiment using the SoSci Survey [34]. The questionnaires collected demographic information (age and gender) and assessed dispositional perspective-taking (subscale of the German version of the Interpersonal Reactivity Scale; IRI; [35]) and autistic traits (Autism-Spectrum Quotient—short version; AQ-k; [36]). 

In addition, subjects completed a shortened version of a mood questionnaire (positive and negative affect schedule; PANAS; [37]) prior to and immediately after the experimental tasks, in which they rated their attentiveness, nervousness, and anxiety on a 5 point Likert Scale ranging from 0 (not at all) to 4 (very much).

### 2.7. Statistical Analysis

In the SOFE task, emotional egocentricity was indicated when a particular emotional expression was rated as happier (or more fearful) when coupled with a situational context that elicited happiness (or fear) to the participant, compared to when it was judged without a preceding situational stimulus. First, a bias score was computed individually for each face stimulus as the absolute difference between emotion ratings in the Other condition (i.e., when faces were presented with a preceding situational context) compared to the Face condition (i.e., when faces were presented without a situational context). Next, bias scores were categorized as either egocentric or non-egocentric on a trial-by-trial basis, depending on whether the Other rating moved towards the Self rating (egocentric) or in the opposite direction (non-egocentric), with respect to the Face ratings. For example, if a participant rated a particular facial emotional expression as 60% happy in the Face condition and as 80% happy in the Other condition, we estimated that the situational context biased the judgment of the emotional expression by 20 points. If the participant rated the same situation as eliciting 90% happiness in the Self condition, this bias would be considered egocentric (i.e., the Other rating moved towards the Self rating, with respect to the Face rating). If the participant rated the situation presented in the Other condition as 40% happy, this bias would be considered non-egocentric (i.e., the Other rating moved away from the Self rating, with respect to the Face rating). Egocentric biases were indicated as positive scores and non-egocentric biases as negative scores. 

Furthermore, we defined the congruency between the facial emotional expression and the emotion elicited by the situational context for each trial of the Other condition. If the situation induced the same emotion as the facial expression, the trial was categorized as congruent. If the emotion of the situation and face differed, the trial was categorized as incongruent. Ratings of the Face condition were used to classify the corresponding facial stimuli as either happy (positive ratings) or fearful (negative ratings). Similarly, the situation was considered to elicit happiness if it was positively rated in the Self condition and fear if the rating was negative. We predicted that facial expressions would be rated as more fearful (or less happy) when preceded by a situation that participants considered fear-triggering and as happier (or less fearful) if presented following a happy situation, as compared to when the facial expressions were rated without a context. Moreover, we expected these biases to be stronger in incongruent trials (i.e., when participants observed a happy expression preceded by an event they experienced as fearful or vice versa).

To test for significant emotional egocentricity biases induced by our novel SOFE task independent of stimulation effects, we first performed a one-sample *t*-test against zero using the average bias score in sham participants. We then analyzed the bias scores of all participants with a three-way ANOVA with the within-subjects factors Congruency (congruent vs. incongruent) and Emotion (happy vs. fearful) and the between-subjects factor tDCS condition (active vs. sham). 

In the Director task, accuracy and reaction time data were analyzed using 2 × 2 ANOVAs with the factors Task condition (experimental vs. control) and tDCS condition (active vs. sham). Accuracy was calculated from both the object selection and movement responses [12].

To evaluate tDCS-related changes in subjects’ current emotional state, ratings of attentiveness, nervousness, and anxiety were analyzed separately using 2 × 2 ANOVAs with the factors Time (T_pre_ vs. T_post_) and tDCS condition (active vs. sham). 

In addition, we explored potential factors influencing the degree of egocentric tendencies independent of the stimulation effects, including perspective-taking tendencies (subscale of the IRI) and autistic traits (AQ-k). Toward this end, we calculated Pearson correlations between individual scores and cognitive egocentric biases (based on the accuracy in the experimental condition of the Director task) or emotional egocentric biases (averaged across SOFE task conditions) in sham participants.

The Greenhouse–Geisser correction was used where applicable and post hoc *t*-tests with Bonferroni correction were performed to characterize the significance effects. All tests were two-tailed, and the significant threshold was set at *p* < 0.05. Statistical analyses were carried out using SPSS Statistics (Version 25.0. IBM Corp.: Armonk, NY, USA).

## 3. Results

### 3.1. Demographics and Control Variables

There were no significant group differences between participants who received active compared to those who received sham tDCS with respect to gender, age, years of education, perspective-taking tendencies (subscale of the IRI), and autistic traits (AQ-k). More detailed sample characteristics are reported in Table 2.

With regard to the mood assessment, ANOVAs revealed a significant main effect of Time on ratings of attentiveness (*F*(1, 45) = 9.33, *p* = 0.004, *η*_p_*^2^* = 0.17) and nervousness (*F*(1, 45) = 8.94, *p* < 0.001, *η*_p_*^2^* = 0.31). Before the experiment, participants felt in general more attentive (*M* = 2.87, *SD* = 0.67) and nervous (*M* = 1.19, *SD* = 1.01) as compared to after the experiment (*M* = 2.53, *SD* = 0.80 and *M* = 0.58, *SD*, = 0.77, respectively). For ratings of anxiety, no main effect of Time was found (*F*(1, 45) = 3.07, *p* = 0.087, *η*_p_^2^ = 0.06) indicating no changes in the level of anxiety after the experiment (*M* = 0.15, *SD* = 0.42) as compared to before (*M* = 0.28, *SD* = 0.50). Importantly, no significant interaction between effects of Time and tDCS condition was found (attentiveness: *F*(1, 45) = 1.19, *p* = 0.282, *η*_p_*^2^* = 0.03; nervousness: *F*(1, 45) = 0.004, *p* = 0.952, *η*_p_*^2^* < 0.001; anxiety: *F*(1, 45) = 1.32, *p* = 0.257, *η*_p_^2^ = 0.03).

### 3.2. SOFE Task Performance

#### 3.2.1. Manipulation Check

For a group-dependent manipulation check of emotion induction in the SOFE task, we compared emotion ratings collected in the Self condition between active and sham participants. No significant differences were found for perceived happy (active: *M =* 47.35, *SE =* 3.11; sham: *M =* 50.25, *SE =* 2.49; *t*(45) = 0.73, *p* = 0.47) and fearful situations (active: *M =* −49.35, *SE =* 2.93; sham: *M =* −49.09, *SE =* 3.62; *t*(45) = 0.06, *p* = 0.96).

#### 3.2.2. Emotional Egocentricity Biases 

The average bias score across all SOFE conditions in sham participants was significantly higher than zero (*M* = 5.09, *SE* = 1.51; *t*_0_ (23) = 3.37, *p* = 0.003), indicating the general occurrence of egocentric biases in our novel paradigm.

The three-way ANOVA of the bias scores revealed no significant main effect of Congruency (*F*(1, 45) = 0.39, *p* = 0.534, *η*_p_*^2^* = 0.009), nor of Emotion (*F*(1, 45) = 0.12, *p* = 0.736, *η*_p_*^2^* = 0.003). These results indicated that no significant differences were found in the magnitude of the biases when judging congruent versus incongruent, and happy versus fearful facial expressions. 

**Figure 2:**
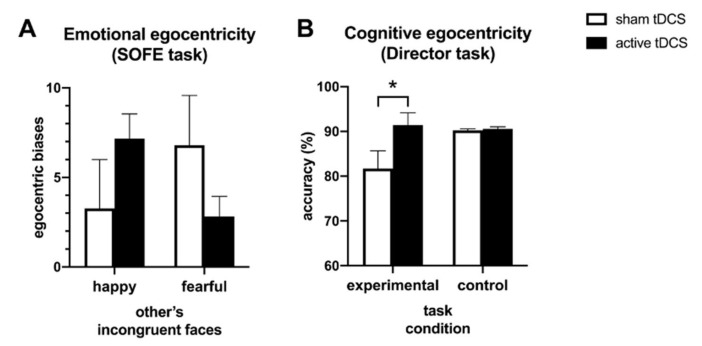
Effects of anodal sham and active tDCS to the rSMG on egocentric biases (mean + SE) assessed in the SOFE and Director task. (**A**) Emotional egocentric biases of the SOFE task were calculated by comparing emotional ratings of others’ facial expressions with a situational, emotion-inducing context to emotional ratings of identical facial expressions without a situational context, with higher scores indicating stronger egocentricity. Biases after active and sham tDCS were plotted for judging other’s happy versus fearful facial expressions. (**B**) Egocentric biases in the Director task were based on the accuracy of switching between two different viewpoints, with higher accuracy indicating lower egocentricity. Biases after active and sham tDCS were plotted for the experimental and control task conditions. * *p* < 0.05.

Regarding general stimulation effects on SOFE task performance, the three-way ANOVA of the bias scores revealed no significant main effect of tDCS condition (*F(*1, 45) = 0.28, *p* = 0.60, *η*_p_^2^ = 0.01). Further, no significant interaction effects were found for tDCS condition × Congruency (*F*(1, 45) = 0.53, *p* = 0.47, *η*_p_^2^ = 0.01) or tDCS condition × Emotion (*F*(1, 45) = 2.87, *p* = 0.097, *η*_p_^2^ = 0.06). However, we found a significant tDCS condition × Emotion × Congruency interaction (*F*(1, 45) = 6.25, *p* = 0.016, *η*_p_^2^ = 0.12). To further explore this three-way interaction, we conducted ANOVAs for congruent and incongruent trials separately. For congruent trials, no significant effects were found (*F < 1)*. For incongruent trials, a significant tDCS condition x Emotion interaction was found (*F*(1, 45) = 7.16, *p* = 0.01, *η*_p_^2^ = 0.14), indicating emotion-specific tDCS effects. As illustrated in Figure 2A, active as compared to sham participants showed lower egocentric biases when judging incongruent fearful faces (active: *M* = 2.82, *SE* = 1.13; sham: *M* = 6.79, *SE* = 2.79) and higher egocentric biases when judging happy faces (active: *M* = 7.16, *SE* = 1.38; sham: *M* = 3.27, *SE* = 2.73). Exploratory analysis of these effects found no significant within-emotion differences between tDCS conditions (fearful, *t*(45) = 1.32, *p* = 0.20; happy, *t*(45) = −1.27, *p* = 0.21). Table 3 lists all mean emotional ratings of the Other and Face conditions in the SOFE task.

### 3.3. Director Task Performance

Due to technical sound issues, 2 subjects (one active and one sham participant) had to be excluded from the Director task analysis. The ANOVA on accuracy revealed a significant tDCS condition x Task condition interaction (*F*(1, 43) = 4.13, *p* = 0.048, *η*_p_*^2^* = 0.088). As illustrated in Figure 2B, this interaction was driven by a significantly higher accuracy on experimental trials for active participants (*M =* 91,40%, *SE =* 2.74) as compared to sham participants (*M =* 81.72%, *SE =* 3.97; *t*(43) = -2.02, *p* = 0.049). In other words, active tDCS enhanced the ability to take the director’s perspective. No significant difference between the tDCS conditions was found in control trials (active: *M =* 90.58%, *SE =* 0.47; sham: *M =* 90.25%, *SE =* 0.35; *t*(43) = -0.57, *p* = 0.57). Regarding reaction times, we found a significant main effect of Task condition (*F*(1, 43) = 20.14, *p* < 0.001, *η*_p_*^2^* = 0.319), indicating that overall, participants responded faster to control trials (*M =* 1.69 sec, *SE =* 0.06) than to experimental trials (*M =* 2.08 sec, *SE =* 0.11). No significant interaction with tDCS condition was found for reaction times (*F*(1, 43) = 3.17, *p* = 0.082, *η*_p_^2^ = 0.069).

### 3.4. Potential Modulators of Egocentric Biases

No significant correlations were found for emotional and cognitive egocentric biases with dispositional perspective-taking (emotional: r = −0.32, *p* = 0.134; cognitive: r = 0.21, *p* = 0.160) and with autistic traits (emotional: r = 0.04, *p* = 0.872; cognitive: r = 0.13, *p* = 0.415). 

### 3.5. tDCS Blinding

With regard to participant blinding, the correct tDCS condition was guessed by about one-third of the participants, which was less than the 50% expected by chance. There was no significant difference of correct guesses between active participants (7 out of 24 guessed correctly) and sham participants (8 out of 23 guessed correctly; χ^2^ = 0.17, *df* = 1, *p* = 0.68), suggesting that our sham protocol was adequate.

## 4. Discussion

This study aimed to investigate emotional egocentricity with a newly developed paradigm, the SOFE task, and to explore underlying neural mechanisms using tDCS. In contrast to existing egocentricity paradigms, in which participants have to rate emotional reactions of others based on contextual information (e.g., visual cues indicating the type of affective touch another person is experiencing but without showing the actual reaction; [8]), we directly confronted participants with others’ facial emotion expressions in response to emotional situations. To increase the ecological validity of our novel paradigm and to measure emotional egocentricity on an individual basis, we confronted participants with emotionally ambiguous situations that could evoke happiness or fear. By applying active or sham tDCS to the rSMG, we further assessed the distinct role of this brain area for emotional as compared to cognitive egocentricity, measured with an established visual perspective-taking task [12,17].

### 4.1. Egocentric Biases in the SOFE Task

Using the SOFE task, we successfully detected emotional egocentric biases independent of stimulation effects. In line with previous research [8,10], we did not find general valence-dependent effects in egocentric tendencies in the absence of tDCS stimulation. More specifically, participants showed similar emotional egocentric biases in trials when confronted with positive (i.e., happy) facial expressions while being in a negative (i.e., fearful) emotional state and vice versa. 

As suggested in our own previous research [15], emotional egocentricity can also be framed in the context of mood-congruency effects by reflecting an over-attribution of the own affective states to others. In fact, there is a large body of evidence showing an enhanced ability to recognize mood-congruent facial expressions [15,38,39,40,41]. However, as previously noted [15], biases in emotion perception as investigated in these studies may be based on more implicit and unconscious processes of self-projection, rather than reflecting abilities in self–other distinction as assessed in classic emotional egocentricity paradigms [8,9,10,11]. In our newly developed SOFE task, participants were instructed to imagine themselves or another person in 24 different emotional situations and to rate their own or others’ emotional states, respectively. Although judging others’ emotions occurred on a perception-based rating of emotional facial expressions, participants had to disengage from their own experience of an emotional situation in order to correctly judge the emotional state of another person. Therefore, egocentric biases in the SOFE task could be interpreted as an index of self–other distinction abilities.

### 4.2. tDCS Effects on Emotional Egocentricity

No overall significant effects of tDCS on emotional egocentricity could be detected. Therefore, our data could not provide conclusive evidence of a general role of rSMG in emotional egocentricity. However, the interaction analysis suggested emotion-specific modulations of egocentric tendencies under tDCS stimulation. Follow-up tests were inconclusive about the direction and nature of the interaction, possibly due to insufficient statistical power (see a priori power analysis). Instead of interpreting the result directly, we examine the data in a purely descriptive way in order to generate new hypotheses to be confirmed in the future. Specifically, active as compared to sham tDCS appeared to have induced descriptively larger differences in egocentric biases across different emotions in the incongruent condition. Although more work is needed before conclusions can be drawn regarding the precise role of rSMG in emotional egocentricity, in the following, we carefully discussed the qualitative patterns based on visual inspection of the data.

Our data suggested lower egocentric biases when judging incongruent fearful faces after active tDCS. Such a finding would be in line with our hypothesis that activity-enhancing anodal tDCS to the rSMG improved the ability to overcome emotional egocentricity, and in line with previous findings that activity-decreasing low-frequency rTMS impaired the ability to overcome emotional egocentricity [8]. However, contrary to our hypothesis, the data seemed to suggest the opposite pattern when judging happy faces in a fearful state, i.e., stronger egocentric biases after active tDCS. Providing a straightforward answer for this effect was difficult. It might be speculated, however, that state-dependent stimulation effects were at work: much evidence had shown that effects of brain stimulation strongly depend on the level of neuronal activity at the time of stimulation [42] and that the manipulation of neural activity prior to the application of brain stimulation could modulate or even reverse the expected effects [43,44]. It might be possible that the induced emotions of happiness versus fear in our SOFE task might have activated the stimulated region to a different degree, and consequently, the level of neural activity at the time of stimulation might have interacted with the actual tDCS effects on egocentricity. Supporting this speculation, a recent study reported the involvement of specific components within the temporoparietal cortex for individual affective states, including happiness and fear [45]. Interestingly, the authors observed that while happiness could be mapped onto the rTPJ in an anterior to posterior arrangement, fear was mapped in an inferior to superior arrangement. In the present study, we applied tDCS to the rSMG, which was located slightly anterior to the rTPJ. Considering the low spatial resolution of tDCS [46], our stimulation might also have affected specifically the adjacent anterior parts of the rTPJ, which was more involved in the processing of happiness than fear [45]. Thus, it could be speculated that the emotion induction of happiness and fear in the SOFE task might have differentially modulated activity in the targeted brain region independent of stimulation.

### 4.3. tDCS Effects on Cognitive Egocentricity

Contrary to our hypothesis, our findings also revealed tDCS effects on cognitive egocentricity. More specifically, participants receiving anodal stimulation of the rSMG showed higher accuracy in experimental trials of the Director task, indicating that tDCS enhanced the ability to switch between two different viewpoints. One possible explanation was that the low spatial resolution of tDCS [46] also influenced the anatomically proximal rTPJ region as well. In previous studies exploring tDCS effects on the same task, it was shown that anodal stimulation of the rTPJ improved visual perspective-taking [12]. Further evidence for the causal role of the rTPJ in switching between the self and other perspective was also found using more focal rTMS [47]. Therefore, the inadvertent stimulation of the rTPJ might account for the unexpected influence on cognitive egocentricity in the present study. 

However, the distinct contributions of rTPJ and rSMG to emotional egocentricity and other socio-cognitive functions were still under debate [1]. Findings of distinct connectivity profiles of the two neighboring regions might point to potential subregions within the temporoparietal cortex subserving specific functions in the context of cognitive and emotional egocentricity [3]. However, it was suggested that rSMG and rTPJ might, in fact, serve the same function but were engaged to a different degree, depending on the emotional versus cognitive content they accessed and regulated [1]. Accordingly, the engagement of the rSMG might only be relevant for specific task constraints in overcoming emotional egocentricity when being in an emotional state oneself [1,8]. Supporting this hypothesis, previous studies investigating emotional or cognitive aspects of empathy in participants in a neutral state found activations in rTPJ but not rSMG [48,49]. Based on our present findings, it could be speculated that distinct emotional states (happy versus fearful) might differentially engage rSMG during emotional egocentricity. Future research should directly compare neural activations during emotional and cognitive egocentricity in participants experiencing different emotional states to further explore the distinct recruitment of rSMG and rTPJ. 

### 4.4. Limitations of the Study 

Our newly developed SOFE task used ambiguous situational stimuli to elicit emotions (Self condition) and asked participants to rate their emotions on a continuum between happiness and fear. Although the ambiguous stimuli implemented in our task had been selected based on a qualitative pretest as causing the most polarized responses on the happiness-fear continuum, we cannot rule out that our stimuli induced other or mixed emotions in some participants, which could not properly be assessed with our rating procedure. Further, we calculated egocentric biases by comparing the emotional ratings of others’ facial expressions with a situational, emotion-inducing context (Other condition) to emotional ratings of identical facial expressions without a situational context (Face condition). The facial stimuli used to rate other’s emotions were identical in the Other and Face condition. Although we used a relatively large number of 48 different faces and the order of the Other and Face condition blocks was counterbalanced across runs, we cannot fully preclude confounding effects of the initial emotion rating task on the following conditions. Furthermore, our participants performed the Director task during tDCS application and the SOFE task immediately afterward. This was done to maximize the effects on emotional egocentricity because it had been shown that anodal tDCS effects during tDCS were less prominent than the after-effects of tDCS [50]. As a result, it was possible that the observed after-effects of tDCS on emotional egocentricity occurred only indirectly, mediated by prior tDCS enhancement while practicing cognitive egocentricity. Given our small sample size, the tDCS effects in the present study should generally be interpreted with caution. 

Moreover, the low spatial resolution of tDCS made it difficult to uniquely attribute the effects to a single region. As discussed above, it was likely that the tDCS-induced electrical field influenced not only the rSMG but also nearby cortical regions. Future research should directly compare focal stimulation of rSMG versus rTPJ using high-definition tDCS (HD-tDCS) or rTMS, ideally in combination with individual SOFE task-based fMRT-guided targeting. This would allow for a proper evaluation of a selective role of the rSMG in emotional egocentricity as well as further investigation of potential emotion-specific modulations. 

## 5. Conclusions

The objective of the current tDCS study was to expand our understanding of emotional egocentricity and rSMG functioning by using a newly developed paradigm, the SOFE task. Our results demonstrated the presence of emotional egocentric biases when directly confronting participants with others’ facial emotion expressions in response to emotional situations. However, our data could not provide conclusive evidence of a general role of rSMG in emotional egocentricity. More research is needed to investigate whether distinct emotions differentially affect egocentricity and to distinguish rSMG from rTPJ activity related to cognitive egocentricity.

## Figures and Tables

**Table 1:** Ambiguous situational stimuli of the SOFE task to induce either happiness or fear. Photographs from a first-person perspective were accompanied by a short written description of the situation.

	Situation
1	Riding down a water chute
2	Giving a speech in front of a huge audience
3	Riding a roller coaster down a steep track
4	A huge dog is approaching
5	A shark is swimming by
6	Arriving alone in a big foreign city
7	Standing close to big waterfalls in the sun
8	Dancing in a crowd to an electronic concert
9	Setting foot on a stage to perform
10	Crossing a rope bridge in a deep jungle
11	Watching a scene of a horror movie
12	A tarantula is crawling over your hand
13	Speeding down a roller coaster
14	Hiking on a snowy trail at the edge of an abyss
15	Sitting in a lonely boat on the glassy sea
16	A shark swims up to the underwater cage
17	Rafting down a mountain river
18	Driving at extreme speed through a tunnel
19	Approaching a big fish on a diving trip
20	Waiting for the roulette wheel to stop spinning
21	Looking downwards from a Ferris wheel
22	Your speech makes the crowd laugh out loud
23	Jumping out of a plane for a skydive
24	Entering a vast desert on a desolate road

**Table 2:** Demographics and individual characteristics.

tDCS Condition	Active	Sham		
N (female)	24 (14)	23 (14)		
	M (SD)	M (SD)	*t* ^a^	*p*
Age (years)	25.9 (2.8)	25.9 (2.8)	0.004	0.965
Years of education	13.5 (0.50)	13.2 (1.2)	1.22	0.231
Perspective-taking (IRI subscale)	15.4 (2.1)	16.0 (2.9)	0.79	0.433
Autistic traits (AQ-k)	8.7 (5.3)	8.6 (5.6)	0.04	0.972

tDCS: transcranial direct current stimulation, M: mean, SD: standard deviation, IRI: Interpersonal Reactivity Scale, AQ-k: Autism-Spectrum Quotient—short version, ^a^ Independent samples: *t*-tests, two-tailed.

**Table 3:** Mean emotional ratings and bias scores for all Other and Face conditions in the SOFE task in dependance of own emotional ratings in the Self condition (Emotion) and their congruence with the Face condition (Congruency). Note that bias scores were categorized as either egocentric (positive scores) or non-egocentric (negative scores) on a trial-by-trial basis.

			Other Condition		Face Condition		Bias Scores	
tDCS	Emotion	Congruency	M	SE	M	SE	M	SE
Sham	Happy	Congruent	41.20	2.77	40.01	2.69	5.66	1.64
	Fearful		−42.84	2.43	−41.72	2.21	4.65	1.07
	Happy	Incongruent	29.85	3.98	33.11	2.23	3.27	2.73
	Fearful		−32.62	3.75	−39.41	2.47	6.79	2.79
Active	Happy	Congruent	39.79	3.50	40.19	3.35	3.06	1.51
	Fearful		−41.28	2.16	−39.64	2.27	3.63	0.98
	Happy	Incongruent	24.76	2.81	31.92	2.47	7.16	1.38
	Fearful		−33.13	2.11	−35.95	1.82	2.82	1.13

tDCS: transcranial direct current stimulation, M: mean, SE: standard error.
